# Systemic and Local Responses to Repeated HL Stress-Induced Retrograde Signaling in *Arabidopsis*

**DOI:** 10.3389/fpls.2012.00303

**Published:** 2013-01-17

**Authors:** Matthew J. Gordon, Melanie Carmody, Verónica Albrecht, Barry Pogson

**Affiliations:** ^1^School of Biochemistry and Molecular Biology, Australian Research Council Centre of Excellence in Plant Energy Biology, Australian National UniversityCanberra, ACT, Australia

**Keywords:** systemic acquired acclimation, high light, photoprotection, retrograde signaling, oxidative stress

## Abstract

Chloroplasts of leaves under high light stress initiate signals to the nuclei of both exposed and distal leaves in order to acclimate against the potential threat of oxidative damage: a process known as high light systemic acquired acclimation (HL SAA). This study explores the nature of HL SAA, synergistic interactions with other environmental stresses, and the impact of repeated HL stress on the acclimation response of exposed and distal leaves. This necessitated the development of novel experimental systems to investigate the initiation, perception, and response to HL SAA. These systems were used to investigate the HL SAA response by monitoring the induction of mRNA in distal leaves not exposed to the HL stress. Acclimation to HL is induced within minutes and the response is proportionally dependent on the quality and quantity of light. HL SAA treatments in conjunction with variations in temperature and humidity reveal HL SAA is influenced by fluctuations in humidity. These treatments also result in changes in auxin accumulation and auxin-responsive genes. A key question in retrograde signaling is the extent to which transient changes in light intensity result in a “memory” of the event leading to acclimation responses. Repeated exposure to short term HL resulted in acclimation of the exposed tissue and that of emerging and young leaves (but not older leaves) to HL and oxidative stress.

## Introduction

Acclimation to changes in the environment is required for optimal plant performance under adverse conditions. Factors such as light, temperature, drought, mineral concentrations, and biotic infection are all capable of causing extensive damage to plants as well as inducing short and long term acclimation responses (Stitt and Hurry, 2002; Durrant and Dong, 2004; Bartels and Sunkar, 2005; Atkin et al., 2006; Gorsuch et al., 2010; Biswal et al., 2011). High light (HL) causes damage to DNA, proteins, and lipids, including components of the photosynthetic apparatus (Kalbin et al., 2001; Takahashi and Badger, 2011). Exposure to prolonged periods of HL increases the generation of reactive oxygen species (ROS) and alters the redox state of photosynthetic components such as the electron carrier, plastoquinone (Karpinski et al., 1997; Asada, 2006). These components provide important retrograde signals that communicate the chloroplast status to the nucleus proving important information to drive transcriptional activation of defense systems (Pogson et al., 2008; Ramel et al., 2012). Recently, evidence for novel HL retrograde signals including the SAL1-PAP pathway and an oxidative by-product of beta-carotene has been published (Estavillo et al., 2011; Ramel et al., 2012).

Chloroplastic and retrograde signaling in response to HL induces (1) pathways that allow for the dissipation of excess energy; (2) systems that detoxify the harmful by-products of HL; and (3) mechanisms that reduce the amount of light absorbed by the plant. Plants have also evolved different mechanisms that facilitate the dissipation of accumulated excess energy absorbed under HL conditions, including chlororespiration, cyclic electron flow (CEF), photorespiration, and non-photochemical quenching (NPQ; Rumeau et al., 2007; Bauwe et al., 2010; de Bianchi et al., 2010; Johnson, 2011). Depending on light conditions, NPQ can account for 50% or more of the absorbed energy (Demmig-Adams et al., 1996) and thus is one of the main avenues for excess energy dissipation under HL exposure. On the other hand, to detoxify accumulating ROS plants can also use enzymes or plant pigments to convert ROS into more benign molecules. Superoxide dismutase (SOD) and ascorbate peroxidase (APX) are responsible for directly detoxifying ROS, superoxide (${\text{o}}_{\text{2}}^{•-}$), and hydrogen peroxide (H_2_O_2_), respectively. In contrast, plant pigments such as carotenoids and tocopherols remove ROS via chemical and physical quenching (Conn et al., 1991; Kobayashi and Della Penna, 2008).

From dawn till sunset plants are subjected to varying light intensities due to the angle of the sun and transient shade from clouds, leaves, and neighboring plants. Living in such an environment creates “hot spots” of solar energy that have the potential to cause extensive local photo-oxidative damage to plants. Moreover, such hotspots can trigger rapid acclimation in tissues directly experiencing high irradiance stress, and in distal tissues still under partial shade (i.e., leaves that do not experience HL stress). Acclimation of metabolism in distal leaves occurs as a result of a 15- to 60-min short term HL exposure, termed high light systemic acquired acclimation (HL SAA), in which HL stressed tissues of individual plants communicate to the distal parts of the plant initiating stress acclimation. Even though research over that last decade has significantly progressed the understanding of HL SAA many unknowns still exist in regards to the identity of the retrograde signal(s) and the acclimation processes which they govern. Also unclear is the exact nature of the synergistic relationships between different stresses, how they affect the initiation of HL SAA and subsequent acclimation processes against multiple stresses (Koussevitzky et al., 2007; Mullineaux and Baker, 2010).

By exposing 1/3 of the *Arabidopsis* rosette to non-specific HL, research has shown that SAA seems to be tightly regulated by retrograde signals initiated through changes in photosynthesis during HL stress, specifically changes to the PQ pool redox state and ROS production (Karpinski et al., 1997; Rossel et al., 2007; Muhlenbock et al., 2008). H_2_O_2_ accumulates rapidly under HL and remains a likely signaling candidate as H_2_O_2_-signaling components have been implicated in triggering HL SAA (Mateo et al., 2004; Muhlenbock et al., 2008; Miller et al., 2009) and are associated with inducing defense responses under both abiotic and biotic stress (Vanderauwera et al., 2005; Miller et al., 2007, 2010; Muhlenbock et al., 2008). Additionally, recent publications suggest the involvement of light-wavelength-specific electrochemical and memory-based signaling systems influenced by both calcium-mediated signaling and glutathione (GSH; Karpinski and Szechynska-Hebda, 2010; Szechynska-Hebda et al., 2010). Nonetheless, specific components and connections between these different processes, particularly from a temporal perspective, remain to be clarified.

Microarray data shows that distal protective mechanisms in response to short term non-specific HL exposure in 1/3 of the *Arabidopsis* rosette are controlled by the transcriptional regulation of many HL-, ROS-, pathogen infection-, hormone-, and drought-responsive genes (Mullineaux et al., 2000; Rossel et al., 2007; Muhlenbock et al., 2008). Among these genes are transcripts responsible for ROS detoxification and signal transduction such as zinc finger transcription factors (ZAT), APXs, and pathogenesis-related proteins (PRs). The induction of these transcripts and subsequent acclimation is known to impart enhanced tolerance to two distinct types of stress: pathogen infection and HL oxidative stress (Rossel et al., 2007; Muhlenbock et al., 2008; Szechynska-Hebda et al., 2010). The relationship between HL SAA, the transcriptional activation of these many genes, their role in specific HL signaling, and acclimation processes however remains less clear.

In addition to short term transient HL SAA the growth of young, unstressed developing leaves can be altered by changing the environment in which the mature leaves are maintained. This process of developmentally linked long term acclimation allows plants to exhibit further phenotypic changes to improve performance of new tissue to that which the mature leaves were exposed; whether through differences in irradiance, CO_2_, or temperature (Yano and Terashima, 2001; Coupe et al., 2006; Gorsuch et al., 2010). These modifications to new leaves include modifying leaf structure, growth rates, leaf and palisade tissue thickness, epidermal cell shape and size, as well as chloroplast number and density in the developing leaves (Lake et al., 2001; Yano and Terashima, 2001; Thomas et al., 2004; Coupe et al., 2006; Miyazawa et al., 2006; Araya et al., 2008; Jiang et al., 2011; Woo et al., 2011). Even though the exact mechanisms and signaling processes from mature leaves to meristems remain elusive there is evidence suggesting the possible involvement of retrograde signaling components such as ROS, the redox status of the PQ pool, other plant hormones, or microRNAs (Yano and Terashima, 2001; Thomas et al., 2004; Coupe et al., 2006; Jiang et al., 2011).

Many questions persist in regards to the mechanisms controlling short term HL SAA, the synergistic relationships with other stresses, and its role in acclimation processes that occur during a single day and over longer periods of time (several days). This is a study in two parts, firstly, the investigation of how light and environmental conditions affect HL SAA and secondly, the study of repeated HL treatments on signaling in exposed and distal mature leaves. This was achieved through (1) the development of a novel treatment system to further characterize the short term HL SAA gene activation in existing tissues under varying ambient qualities such as the duration of treatment, light intensity, temperature, and relative humidity (RH), as well as to determine the spatial distribution of oxidative stress tolerance across the rosette; (2) investigation of whether and how repeated, transient, and localized HL treatments can alter acclimation responses within existing mature leaves.

## Materials and Methods

### Growth conditions and light experiments

For all experiments *Arabidopsis thaliana* (Col-0) plants were cultivated in soil under a 12-h photoperiod of 150 ± 25 μmol photons m^−2^ s^−1^, 23/22 ± 2°C day/night temperatures, and 70 ± 10% day/night RH. All HL treatments utilized a new light emitting diode (LED)-array system and mature (approximately 4 weeks old) *Arabidopsis* plants. *Arabidopsis* leaf position for tissue collection were counted according to *Arabidopsis* phyllotaxy (Jurgens, 2001).

**Figure 1:**
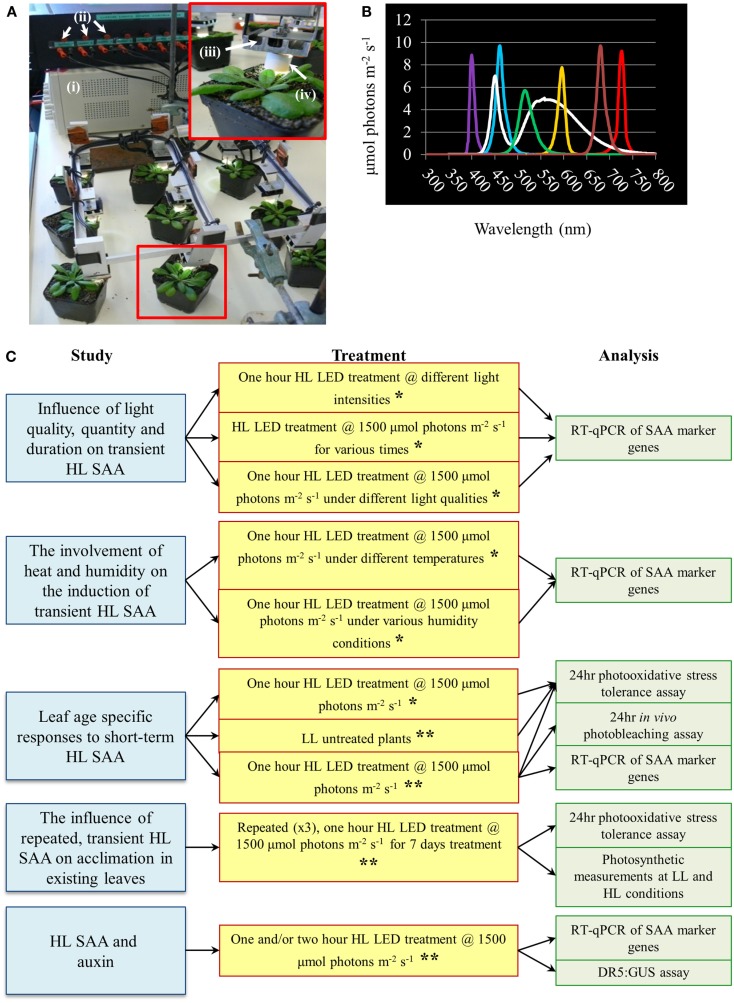
**High light systemic acquired acclimation-array, spectral output of specific LEDs and schematic overview of experiments conducted throughout the research article**. **(A)** HL LED-array. Inset depicts a close-up image of a single treated *Arabidopsis* plant. Components of the HL LED-array include (i) power supply, (ii) current limiters for each LED, (iii) individual movable LED stage with heat sink, and (iv) light focusing lens. **(B)** Measured irradiance spectra from colored LEDs, *n* = 3. **(C)** Schematic diagram of the main areas of study, light treatments, and methods of analysis conducted throughout the research article. For all light treatment mature plants of approximately 4 weeks old grown under normal light conditions as detailed in Section “Materials and Methods” are used. For more detail on each individual treatment and analysis refer to Section “Materials and Methods.” *Experiment conducted irrespective of leaf position. **Experiment conducted taking leaf position in to account.

The HL SAA LED-array system consisted of nine white Luxeon III star LEDs (Lumileds Lighting)^1^ controlled by current limiters and focusing lenses which produced a light spot with 1 cm radius (Karpinski et al., 1999; Rossel et al., 2007; Muhlenbock et al., 2008; Szechynska-Hebda et al., 2010). For initial HL treatments, HL LED-array validation and HL SAA transcriptional analysis, individual leaves of nine plants were simultaneously exposed to HL (1500 ± 50 μmol photons m^−2^ s^−1^) or to LL conditions (40 ± 25 μmol photons m^−2^ s^−1^). Subsequently, HL, control, and distal tissues from three treated individual plants were pooled to yield three “biological” replicates per tissue, immediately frozen in liquid N_2_, and stored at −80°C. During analysis of environmental effects on HL SAA, plants were subjected to: HL exposure (1500 ± 50 μmol photons m^−2^ s^−1^) for either 5, 30, 60, and 120 min; varied irradiances of 250, 500, 1000, or 1500 ± 50 μmol photons m^−2^ s^−1^ for 60 min. Light quality treatments were performed with white, ultra violet A (400 nm), blue (460 nm), green (515 nm), yellow (600 nm), red (680 nm), and far-red (720 nm) specific light. The wavelength and irradiance of the specialized LEDs (Roithner LaserTechnik, Vienna, Austria) was verified by a spectrophotometer (Figure 1). For the repeated medium-term treatments, mature *Arabidopsis* plants were either subjected to HL array treatment (1500 ± 50 μmol photons m^−2^ s^−1^) three times a day for 60 min (separated by 120 min), for eight consecutive days, or remained untreated in the same growth environment.

All variable humidity and temperature specific HL SAA treatments were performed in a controlled environment chamber (Conviron S10H, Conviron, Ltd., Winnipeg, MB, Canada). Plants were subjected to either HL LED-array exposure (1500 ± 50 μmol photons m^−2^s^−1^) under varied humidity levels (30, 55, and 90% RH) at 21°C or at increasing temperatures (21, 28, and 32°C) at 55% RH.

### RT-qPCR analysis

For gene transcript analysis, RNA was extracted from frozen samples using an RNeasy Plant Mini Kit (Qiagen, Ltd.) as instructed by the manufacturer’s instructions. RNA was converted to cDNA using SuperScript III Reverse Transcriptase (Invitrogen), and RT-qPCR performed using a LightCycler 480 (Roche) with either the LightCycler Universal ProbeLibrary or Sybr green specified by the manufacturer’s instructions. The LightCycler 480 software application (Roche; version 1.5.0) was used to determine crossing-point values for each reaction, amplification efficiency of each primer set, validation of each reaction, and relative expression values obtained as described (Pfaffl, 2001). For initial RT-qPCR experiments and primer validation target transcript levels were normalized to both reference genes, *CYCLOPHILIN 5* *(CYP5)* and *PROTEIN PHOSPHATASE 2A (PP2A)*. In subsequent experiments target transcript level were normalized to one of the aforementioned reference genes. List of primers are outlined in Table 2. Statistical significance of results was tested by conducting paired student *t*-tests (between LL controls and other samples) and one-way analyses of variance (ANOVA) on all samples using the scientific statistical analysis program, SigmaPlot12 (Systat Software, Inc.). Least significant difference (LSD) *post hoc* tests were used where one-way ANOVAs indicated significant differences between factors.

### *In vitro* and *in vivo* stress tolerance assays and photosynthetic measurements

The *in vitro* photo-oxidative stress tolerance assay was performed after short term HL SAA treatments and at the end of the 8-day period of the repeated, HL SAA study, using a method adapted from Rossel et al. (2007). Leaf disks from each of the treated plants were removed and floated (abaxial side down) on 0.5 M H_2_O_2_ in a clear 200 μL 96-well plate. The leaf disks were then exposed to HL (1500 ± 50 μmol photons m^−2^ s^−1^) for 60 min and moved to LL growth conditions for 24 h while remaining in H_2_O_2_ solution, during which time photographs were taken periodically every 2 h to determine the extent of bleaching of the leaves. Analysis of the photos was performed using ImageJ^2^ and Microsoft Excel (Microsoft, Washington, USA). The percentage of healthy (green), and bleached (white) tissue in each leaf disk was calculated and compared over time.

Plants subjected to the *in vivo* HL-stress tolerance assay were exposed to the HL spot treatment (leaf 4 @ 1000 ± 50 μmol photons m^−2^ s^−1^) for 60 min or remained untreated under LL growth conditions. Following the initial HL SAA treatment, treated and untreated whole plants were placed in a controlled growth environment chamber (Conviron S10H, Conviron, Ltd., Winnipeg, MB, Canada) under HL growth conditions for 24 h (1500 ± 25 μmol photons m^−2^ s^−1^, 23/22 ± 2°C). Plants remained well watered for the duration of the 24-h treatment. During the 24-h HL treatment photographs were taken periodically to assess the first appearance photobleaching.

Chlorophyll fluorescence measurements were taken during the repeated, HL SAA study using an IMAGING-PAM chlorophyll fluorometer and analyzed with the ImagingWin software application (Walz, Effeltrich, Germany) as described (Krause and Weis, 1991; Oxborough, 2004; Baker, 2008). Tissue was sampled from existing mature tissues, as well as from the treated leaf as outlined in the transient HL SAA experiments.

### Microarray data comparison and transcript analysis

Microarray data from Rossel et al. (2007) was directly compared to six different studies of auxin microarray experiments (Sawa et al., 2002; Zhao et al., 2003; Redman et al., 2004; Overvoorde et al., 2005; Nemhauser et al., 2006; Lee et al., 2009). Only gene transcripts that demonstrated significant changes in gene expression (as determined in each respective article) were considered in this comparison. The functional characterization was based on gene ontology (GO) descriptions available on TAIR 10 (2012). As all transcripts had numerous GO descriptions, preference was given to auxin-related or stress networks.

### Auxin detection using DR5:GUS transgenics

*DR5:GUS* transgenic lines were provided by Dr. Christopher Cazzonelli (ANU). Mature *DR5:GUS* transgenic plants were either treated with LL conditions (40 ± 25 μmol photons m^−2^ s^−1^), or with HL spot (1500 ± 50 μmol photons m^−2^ s^−1^) for 60 or 120 min. GUS staining and localization was performed using a modified version of the GUS visualization assay (Stomp, 1992). Each plant was divided into separate 2 ml microfuge tubes.

## Results

### Transcriptional regulation of genes specific to HL SAA

A new HL SAA LED-array system was developed to enable repeated exposure of a leaf without altering the growth conditions of other leaves (Figure 1). This treatment applied a spot of light in the absence of heat and shading, facilitating a more environmentally relevant and specific test of HL-induced SAA compared to previous light treatment methods (Karpinski et al., 1999; Rossel et al., 2007; Muhlenbock et al., 2008; Szechynska-Hebda et al., 2010). Due to the specific nature of the treatment it also allows differentiation between retrograde signals derived from other stresses and solely HL.

It was necessary to validate the new method given the differences in light regimes between this and previous HL and HL SAA treatments. Thus, a detailed analysis of transcript changes in response to HL spot treatment of numerous genes involved in a range of plant processes from light signaling to ROS metabolism was performed. This provided (1) a greater understanding of the genetic regulation of HL retrograde responses governing the initiation and perpetuation of SAA; (2) identification of SAA marker genes that could be used in this study for an efficient quantification of HL SAA activation under different treatment regimes; and (3) identification of genes which are specifically induced in distal leaves, but not exposed leaves, which may give novel insight into the mechanisms or role of HL SAA in *Arabidopsis*.

For this analysis, genes were selected based on one of two factors: if they were reported to be involved in HL or SAA in previous studies (Karpinski et al., 1999; Mullineaux et al., 2000; Rossel et al., 2002, 2007); and according to their relative importance and involvement in light and stress signaling pathways in *Arabidopsis* (Rapp and Mullet, 1991; Yamaguchi-Shinozaki and Shinozaki, 1993; Braam et al., 1997; Hutin et al., 2003; Barrero et al., 2006; Lee et al., 2007; Zhu et al., 2010). Over 45 transcripts induced in both HL and distal leaf tissue, as well as genes reported to be induced in distal, but not HL-exposed leaves, were chosen for confirmation with RT-qPCR (Table 1). The vast majority of genes reported to be induced by HL SAA in microarrays and other experiments when a 1/3 of the rosette is exposed to HL were confirmed to be induced by the single spot of the HL LED-array system. However, all except one of the reported SAA specific inducible genes were also induced in the exposed tissue. The single gene that exhibited distal-specific expression, *Gretchen Hagen* *3.3* (*GH3.3*; Table 1), encodes an enzyme involved in maintaining auxin homeostasis by auxin conjugation with amino acids (Staswick et al., 2005), which will be addressed later in this manuscript. *REDOX RESPONSIVE FACTOR 1* (*RRTF1*) and *ZAT10* were selected as marker genes for HL SAA in subsequent experiments as both transcripts exhibited strong, relatively consistent transcript induction under short term HL spot treatment.

**Table 1:** **Analysis of gene transcript abundance after 60 min HL LED-array treatment**.

Gene locus	Annotation (TAIR10)	**HL** mRNA fold change	Standard error	**DL** mRNA fold change	Standard error	Ref*
AT1G43160	*RAP2.6*	**2395**	1606	**450.4**	253.1	*ii*
AT4G15210	*ATBETA AMY*	**1090.3**	277.3	**85.7**	9.9	*i, iii*
AT3G22840	*ELIP1*	**79.1**	8	**7.3**	1.7	*ii*
AT4G34410	*RRTF1*	**68.9**	13.8	**28.2**	5	*iii*
AT5G20230	*BCB*	**39.8**	7.3	**65.8**	17.5	*iii*
AT4G28140	*Unknown (F26K10.20)*	**34.8**	6.8	**10.4**	2.5	*iii*
AT1G52890	*ANACO19*	**31.6**	12.6	**10.5**	0.8	*iii*
AT3G63060	*EDL3*	**29.5**	6.7	**4.9**	0.5	*iii*
AT1G12610	*DDF1*	**21.2**	3.9	**10.3**	3.1	*iii*
AT5G05410	*DREB2A*	**15.5**	1.5	**4.2**	0.6	*i, ii*
AT1G02400	*DTA1*	**12.2**	1.7	**9**	2.6	*iii*
AT1G28370	*ERF11*	**10**	4	**8.3**	2.2	*iii*
AT5G59820	*ZAT12*	**9.9**	1.4	**11.2**	0.4	*i*
AT5G04340	*ZAT6*	**9.3**	3	**3.8**	0.3	*iii*
AT1G01480	*ACS2*	**8.6**	0.8	**7.1**	1.7	*iii*
AT5G67300	*MYB44*	**8.4**	0.4	**4.9**	1.7	*iii*
AT2G42360	*Putative zinc finger protein*	**8.2**	2.6	**4.5**	1.2	*iii*
AT1G27730	*ZAT10*	**7.6**	0.8	**6.2**	1.9	*i*
AT1G21550	*Put. calcium binding protein*	**6.6**	0.9	**3.7**	0.4	*iii*
AT2G38870	*PR6-like*	**5.9**	2.5	**8.7**	3.2	*iii*
AT4G18170	*WRKY28*	**5.5**	0.5	**4.3**	0.4	*iii*
AT3G46660	*UGT76E12*	**5.4**	1.2	**2.2**	0.4	*iii*
AT5G47220	*ERF2*	**5.2**	1.6	**6.5**	1.4	*iii*
AT3G14440	*NCED3*	**3.8**	0.4	**4.3**	1.4	*ii*
AT2G35980	*NHL10*	**3.8**	0.9	**2.2**	0.3	*iii*
AT4G35090	*CAT2*	**3.3**	0.4	**2.5**	0.2	*i*
AT4G21680	*NRT1.8*	**2.6**	0.4	**3.6**	0.4	*iii*
AT5G50760	*Unknown (MFB16.16)*	**2.2**	0.1	**2.8**	0.5	*iii*
AT2G23170	*GH3.3*	**1.3**	0.5	**6.2**	0.9	*iii*
AT2G47730	*GST6*	**2.6**	0.3	**2.12**	0.2	*ii*
AT5G57560	*TCH4*	**2.2**	0.2	**2.1**	0.3	*ii*
AT5G52310	*RD29a*	**2**	0.5	**1.9**	0.2	*ii*
AT3G09640	*APX2*	**258.5**	234.2	**1.2**	0.6	*ii*
AT4G14690	*ELIP2*	**100.5**	18.4	**0.7**	0.1	*ii*
AT1G67090	*RBCS1A*	**2.2**	0.1	**1.1**	0.7	*ii*
AT2G22470	*AGP2*	**1.9**	0.2	**1.6**	0.2	*iii*
AT1G17170	*GST24*	**1.8**	0.6	**0.9**	0.1	*iii*
AT2G31570	*GPX2*	**1.7**	0.4	**1.2**	0.2	*i*
AT1G05680	*UGT74E2*	**1.4**	0.1	**0.9**	0.2	*iii*
AT1G29910	*LHCB1.2*	**1.3**	0.1	**1.4**	0.2	*ii*
AT2G27030	*CAM5*	**1.1**	0.1	**1.1**	0	*ii*
AT4G33630	*EX1*	**1.1**	0.1	**1.4**	0.1	*ii*
AT3G57260	*PR2*	**0.7**	0.4	**0.8**	0.3	*ii*

*References used for gene selection: i. Karpinski et al. (1999), Mullineaux et al. (2000), Rossel et al. (2007), Rossel et al. (2002), ii. Rapp and Mullet (1991), Yamaguchi-Shinozaki and Shinozaki (1993), Braam et al. (1997), Hutin et al. (2003), Barrero et al. (2006), Lee et al. (2007), Zhu et al. (2010), iii Rossel et al. (2007)*.

*Bold indicates gene fold change*.

### Influence of light quality, quantity, and duration on transient HL SAA

**Figure 2:**
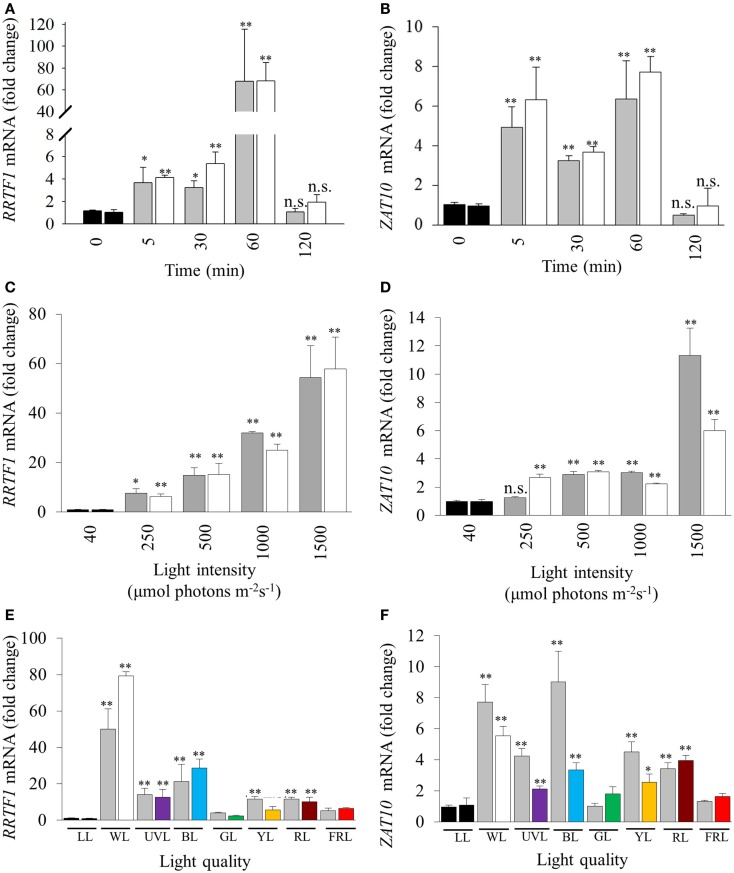
**Influence of duration, intensity, and quality of HL on SAA-mediated gene expression**. **(A)**, **(C)**, and **(E)** *RRTF1*; **(B)**, **(D)**, and **(F)** *ZAT10*. LL (black bars, 40 ± 25 μmol photons m^−2^s^−1^), HL-treated leaves (white, 1500 ± 50 μmol photons m^−2^s^−1^), and distal leaves (grey). **(A,B)** Duration of HL (1500 μmol photons m^−2^ s^−1^) treatments after 0, 5, 30, 60, 120 min; **(C,D)** 60 min HL at 250, 500, 1000, and 1500 ± 50 μmol photons m^−2^s^−1^; **(E,F)** 60 min HL (1500 μmol photons m^−2^ s^−1^) using LEDs of different light qualities: LL control plants (black, LL), distal (grey), white HL (WL), ultra violet light (UVL), blue (BL), green (GL), yellow (YL), red (RL), and far-red (FRL). For each sample *n* = 6, *p < 0.005, **p < 0.001, n.s., not significant, error bars indicate standard error. LSD *post hoc* tests from one-way ANOVAs show that for both *RRTF1* and *ZAT10* plants under white and blue light LED treatments caused the significant SAA gene induction (*P* < 0.05). For spectral details, Section “Materials and Methods” and Figure 1.

Light intensity, quality, and duration of exposure all influence the generation of retrograde signals that in turn influence and activate different developmental and acclimation responses of treated tissues (Franklin and Whitelam, 2004; Li et al., 2009). Thus a series of experiments was conducted to explore the relationship between specific light qualities and specific HL SAA (Figure 1C). SAA induction was analyzed in plants treated with HL for 5, 30, 60, and 120 min, respectively. Both *ZAT10* and *RRTF1* transcript levels increased significantly within 5 min in both HL-treated and distal tissues and remained elevated for at least 60 min (Figures 2A,B), largely confirming earlier findings using different HL systems. However, after 2 h of HL exposure, transcript levels declined to pre-HL levels in both treated and distal tissues. Even though both SAA marker genes exhibit significant gene induction within 5 min (fourfold), RRTF1 mRNA accumulation was highest after 60 min (60-fold). Since treatment for 60 min showed the maximal increase in both marker genes, this treatment time was applied for all subsequent analyses.

Previous studies analyzed SAA-induced gene expression using relatively high, and ultimately damaging, light intensities in the order of 1500 μmol photons m^−2^ s^−1^ (Karpinski et al., 1999; Rossel et al., 2007; Muhlenbock et al., 2008). With such elevated light intensities to what extent this reflected severe photo-oxidative stress or changes in the electron transport rate and redox poise could not be evaluated. Consequently, 60 min HL LED-array treatments were performed on *Arabidopsis* plants at different light intensities (250, 500, 1000, and 1500 μmol photons m^−2^ s^−1^, respectively). *RRTF1* and *ZAT10* were induced at already relatively small changes in light intensity (Figures 2C,D). Significantly, treatment with 250 μmol photons m^−2^ s^−1^ was sufficient to significantly increase mRNA-levels for both genes in HL-treated and distal tissues. As the light intensity increased, *RRTF1* transcript levels in both HL and distal tissues increased proportionally. In contrast, *ZAT10* showed a relatively small but significant increase in gene expression in both HL-treated and distal tissues under light intensities lower than 1500 μmol photons m^−2^ s^−1^.

Light quality (wavelength) also plays an important role in HL response, acclimation, and plant developmental processes (Li et al., 2009). The HL-specific SAA response to different wavelengths was investigated using colored LEDs. The white LEDs exhibited two maximum peaks of emission (460, 570 nm). Each colored LED had a single specific peak of wavelength irradiance, namely: UVA (400 nm), blue (460 nm), green (515 nm), yellow (600 nm), red (680 nm), and far-red (720 nm; Figure 1). The relative expression levels of *RRTF1* and *ZAT10* were analyzed after HL treatment under the different light qualities using various statistical tests: independent *t*-tests for each time point, and LSD *post hoc* tests from one-way ANOVA combining all tissue-light treatment combinations. As expected, independent samples *t-*tests for each light quality treatment showed white light caused the most statistically significant SAA gene induction, followed by blue light, while UVA, yellow, and red light had less prominent induction of both *ZAT10* and *RRTF1*(Figures 2E,F). LSD tests from one-way ANOVA reveal that for both *RRTF1* and *ZAT10* white light caused the most prominent SAA gene induction (*P* < 0.05). Under blue light *RRTF1* also shows significant induction in comparison to the majority of the other light treatments (*P* < 0.05). On the other hand the significance of transcriptional changes of *ZAT10* between different light qualities becomes less apparent due to small relative fold changes and experimental variance. The results clearly demonstrate that the degree of HL SAA induction of the marker genes is wavelength-dependent.

### The involvement of heat and humidity on the induction of transient HL SAA

**Figure 3:**
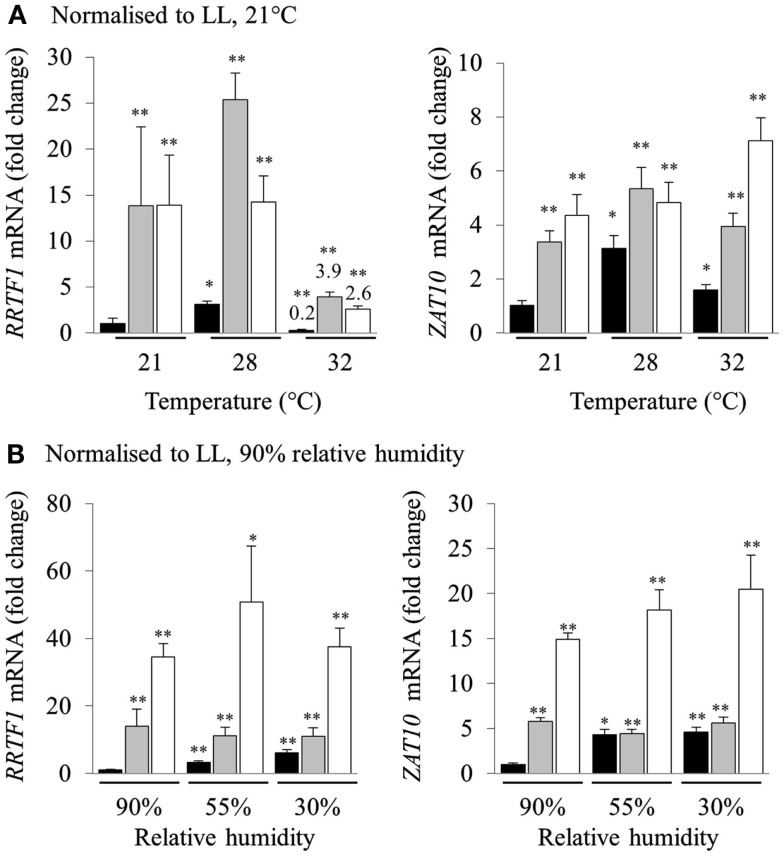
**Analysis of RRTF1 and *ZAT10* transcripts under 21, 28, and 32°C and 90, 55, and 30% relative humidity during HL SAA**. LL control (black), HL (white), and distal (grey). In **(A)** the data are normalized to LL 21°C, for each sample, for **(B)** the data are normalized to LL 90% relative humidity, for each sample *n* = 6, *p < 0.005, **p < 0.001, n.s., not significant, error bars indicate standard error. LSD *post hoc* tests from one-way ANOVAs show that for both *RRTF1* and *ZAT10* plants under 55 and 30% humidity LED treatments the differences between LL and DL tissues is not statistically significant (*P* > 0.05).

High light stress in a natural environment rarely occurs without changes in temperature and humidity, both of which are also powerful inducers of separate retrograde signaling and acclimation defense responses (Fryer et al., 2003; Zhou et al., 2004; Allakhverdiev et al., 2008). Consequently, we investigated the effect of heat and humidity in the induction of HL SAA. Relative transcript levels were normalized to LL at 21°C for each temperature (Figure 3A). Both *RRTF1* and *ZAT10* transcript levels increased after HL exposure in both treated and distal tissues under all analyzed temperatures. Interestingly, expression of *RRTF1* at 28°C was already increased in untreated LL plants compared to LL 21°C. In contrast, at 32°C *RRTF1* showed a significant reduction of transcript levels in all tissues. At the same time, *ZAT10* exhibited a slightly more linear response to HL SAA and heat. The results demonstrate that while the ambient temperature has a significant effect on the induction of HL SAA marker genes HL SAA still occurs at elevated temperatures.

To assess the role of humidity in the induction of HL SAA, different humidity levels were used (30, 55, and 90% RH). Normalizing transcript levels of HL spot exposed plants to LL 90% RH revealed that humidity directly affects the induction of HL SAA. Even though independent samples *t-*tests for each treatment show statistically significant differences between LL samples at 90% humidity, LSD tests on one-way ANOVAs on all sample groups reveal that the difference between the expression of both marker genes under lower levels of humidity in LL and DL tissues is not statistically disparate (*P* > 0.05; Figure 3B). This is especially apparent at 30% RH, where the ability to induce distal expression of both *RRTF1* and *ZAT10* is almost abolished. Therefore, both humidity and temperature have an impact on the induction of HL SAA in distal leaves; with low humidity largely abolishing HL SAA compared to untreated, low humidity exposed plants.

### Leaf age specific responses to short term HL SAA

**Figure 4:**
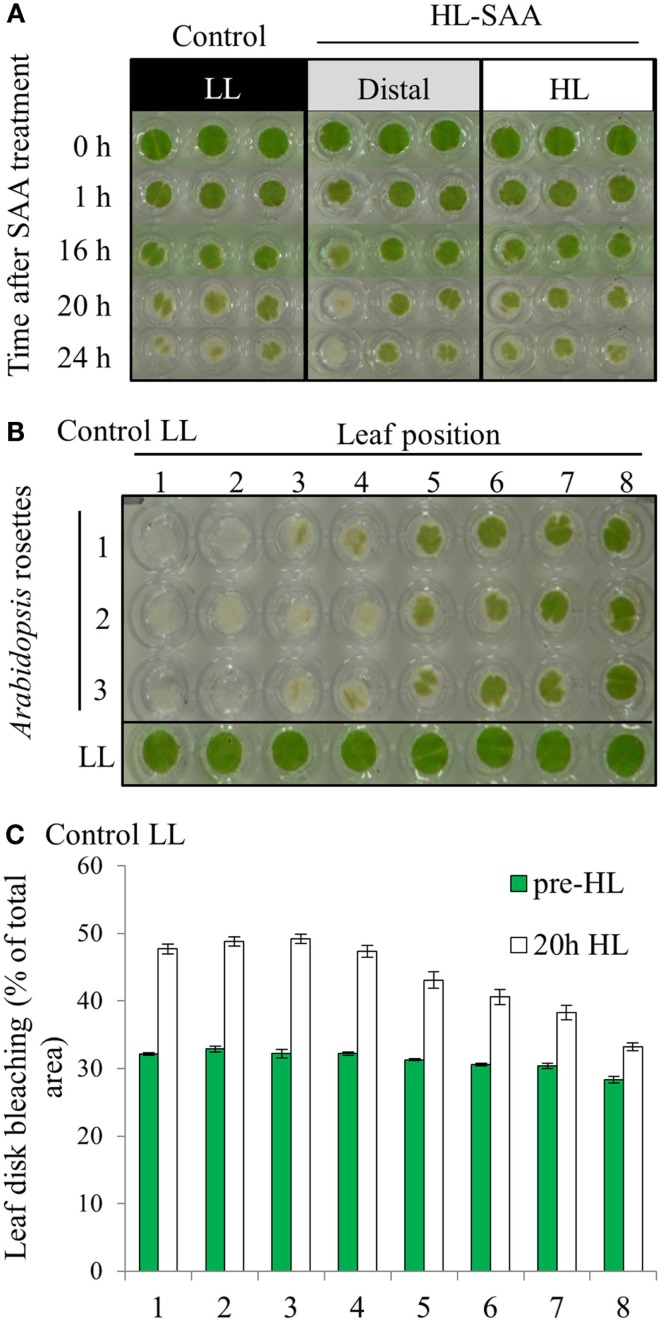
***In vitro* photo-oxidative tolerance of leaf disks during HL SAA and control LL conditions**. **(A)** Representative image of leaf disks sampled from LL (black), HL spot treated leaves (white), and distal leaves (gray). **(B)** Leaf disk assay of basal resistance to photo-oxidative stress across the *Arabidopsis* rosette in leaves under control LL growth conditions. **(C)** Analysis of **(B)** using ImageJ software to distinguish between bleached (white) and unbleached (green) tissues. Plants were either treated to HL spot treatment or remained under LL condition, as explained in Section “Material and Methods.” Following treatment leaf disks were floated on H_2_O_2_ (0.5 M) in a 96-well plate, HL-treated for 60 mins, then returned to 150 μmol photons m^−2^ s^−1^ for 24 h. Photographs were taken throughout the 24-h period, experiment was performed in triplicate, *n* = 3.

In a prior study, treating 1/3 of the rosette with HL for 60 min increased the tolerance to H_2_O_2_-mediated bleaching of leaf disks (Rossel et al., 2007). The new treatment system however exposes a much smaller area of a single leaf with HL, and thus a preliminary investigation into whether this has an impact on the HL SAA physiological response was assessed. The capacity of plant tissues to resist oxidative damage was measured by conducting an *in vitro* photo-oxidative stress tolerance assay which determines the degree of bleaching in response to HL and exogenous H_2_O_2_ (Förster et al., 2005; Rossel et al., 2007). As described in the Section “Materials and Methods” this assay uses HL and H_2_O_2_ as powerful reducing agents to extenuate and rapidly cause oxidative damage to plant tissues, thus inducing pigment bleaching. The extent and the rate at which bleaching occurs can thus be used to estimate the extent of photo-oxidative stress tolerance in plant tissues. However, variability between replicates was greater than variability between treatments (Figure 4A).

**Figure 5:**
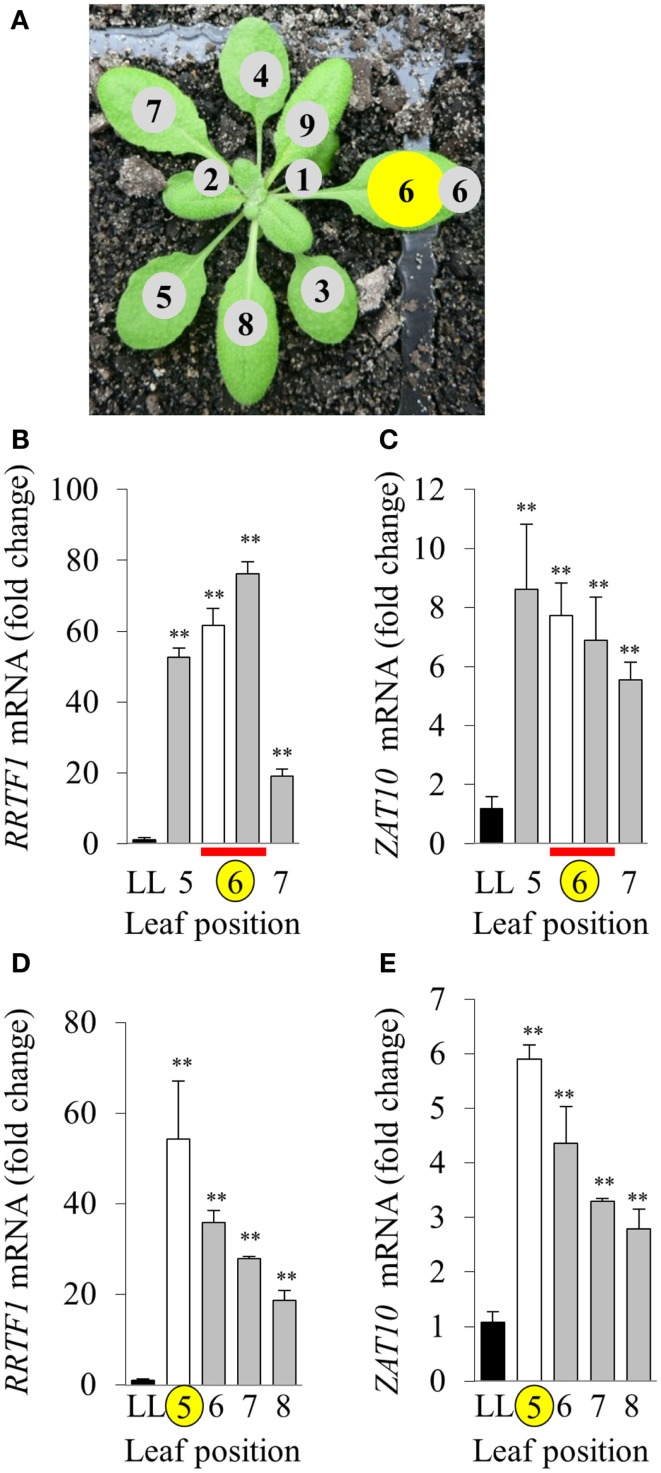
**Leaf position-dependent analysis of RRTF1 and *ZAT10* transcript abundance during HL SAA**. **(A)** Representative image of leaf positions on the *Arabidopsis* rosette, where leaf position 6 is HL-treated (yellow). **(B,C)** HL and distal tissue sampled from leaf 6, distal from leaf positions 5 and 7, where LL control (black), HL (white), and distal (grey) leaves, HL and distal sampled from same leaf (red bar), HL-treated (yellow circle). **(D,E)** HL-treated leaf position 5, distal leaves 6, 7, and 8. *p < 0.005, **p < 0.001, n.s., not significant, error bars indicate standard error. *n* = 6. For both *RRTF1* and *ZAT10* LSD *post hoc* tests on one-way ANOVAs show significant differences between Leaf 5 and 7 when leaf 6 is treated with HL **(B,C)** and also between leaf 5, 6, and 8 when leaf 5 is treated [**(D,E)**; *P* < 0.05].

The variability between leaf disks was hypothesized to be a result of sampling leaves at different developmental stages. Indeed, an *in vitro* oxidative stress tolerance assay investigating leaf positional effects across an *Arabidopsis* rosette under normal LL growth conditions indicated basal leaf age-dependent tolerance in younger leaves (Figures 4B,C). Consequently, leaf age-dependent HL SAA transcriptional responses in the exposed and adjacent leaves were measured. Mature, fully expanded leaf 6 (Figures 5A–C) or 5 (Figures 5D,E) were also exposed to the HL LED-array, and the distal response quantified in two ways: tissue was either sampled from within the same leaf, immediately above (younger) and below (older; Figures 5B,C), or sampled only from the three younger leaves (Figures 5D,E). Independent samples *t-*tests for each leaf show statistically significant induction of the two marker genes in all treated tissues compared to LL controls (Figures 5B–E). More specifically, LSD tests on one-way ANOVAs combining all tissues show significant differences between leaf 5 and 7 when leaf 6 is treated with HL (*P* < 0.05; Figures 5B,C) and also between leaf 5, 6, and 8 when leaf 5 is treated (*P* < 0.05; Figures 5D,E). Thus revealing that in general, distal tissue within the treated leaf, or immediately adjacent, had comparable accumulation of transcripts to the exposed leaf, whereas transcript levels then decreased consistently in progressively younger leaves.

**Figure 6:**
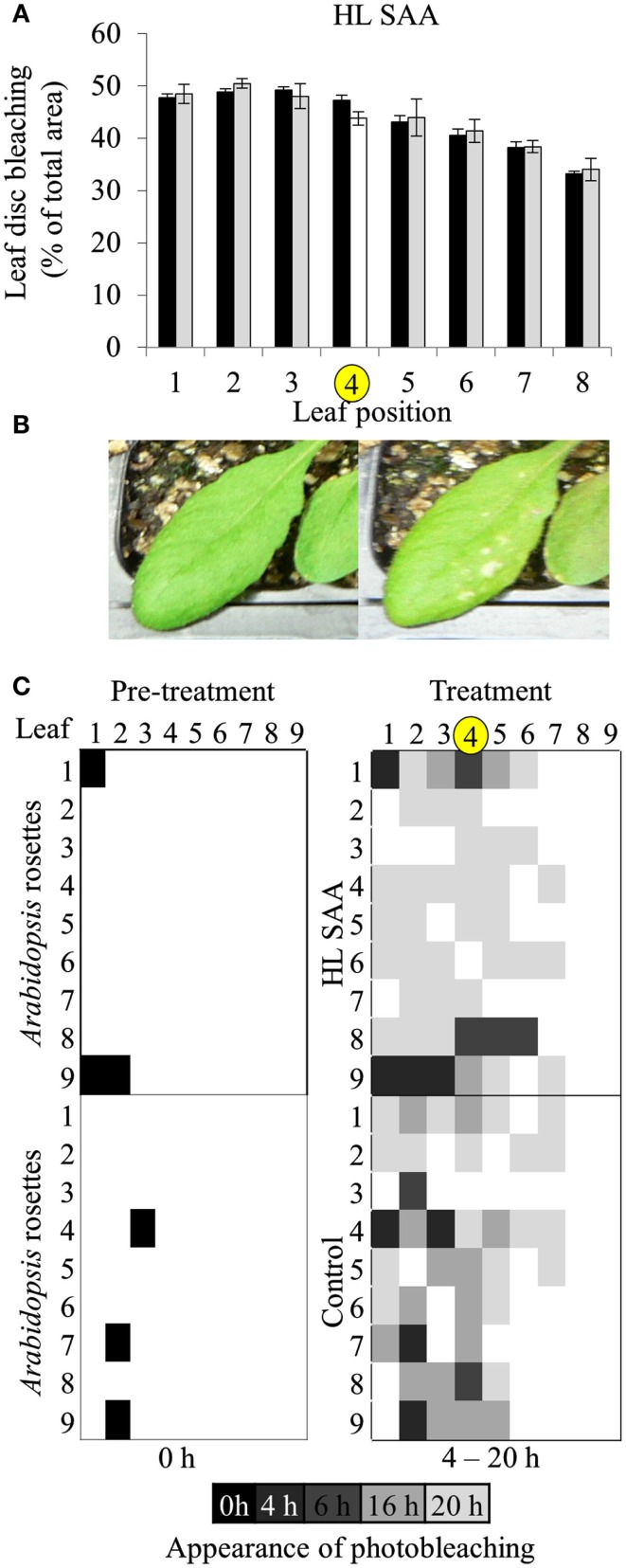
**Leaf position-dependent oxidative stress tolerance during HL SAA**. **(A)** *In vitro* photo-oxidative stress tolerance leaf disk assay, *Arabidopsis* plants were either HL SAA treated at leaf position 4 (white bar, yellow circle) or remained untreated (black). Leaf disks were then taken from all leaves, including distal (gray), floated on H_2_O_2_, and assayed as in Figure 5. Photographs of the leaf disks taken after 20 h and were analyzed using ImageJ software to calculate the percentage of healthy and bleached tissue, *n* = 3. **(B)** First signs of photobleaching during the *in vivo* assay. **(C)** *In vivo* temporal assay of photobleaching development based on leaf position comparing HL SAA treated (top panels) and non-acclimated control plants (lower panels), *n* = 9.

Based on the results of Figures 4 and 5, oxidative stress tolerance was investigated using the *in vitro* photo-oxidative tolerance assay taking leaf position into account (Figure 6A). Leaf 4 was treated with HL spot, leaf disks sampled from all leaves and the assay performed as described in Section “Materials and Methods.” Results from this *in vitro* assay did not indicate any substantial difference in photobleaching development between HL SAA acclimated and non-acclimated plants; however, there was a general trend of increased oxidative tolerance in younger tissues (Figure 6A).

Age-dependent HL SAA was then investigated *in vivo* by determining the first appearance of photobleaching in intact leaves subject to continuous HL after the HL spot treatment. This treatment was used to determine whether there is a specific spatial and age-dependent pattern of the onset of photobleaching as a result of HL SAA. *Arabidopsis* plants were either treated with HL SAA or left non-acclimated. The entire rosette was then subjected to 20 h HL and appearance of photobleaching recorded after 0, 4, 6, 16, and 20 h (Figures 6B,C). In both treated and untreated plants there was less and a slower rate of induction of bleaching in younger leaves. Interestingly, the temporal aspect of this assay revealed a slight difference between HL SAA acclimated and control, non-acclimated plants in that most HL SAA plants developed photobleaching at 20 h, whereas photobleaching in control plants appeared more rapidly and sporadically across the rosette under HL (indicated by increased number of darker shaded boxes). The temporal aspect of this assay indicates that HL SAA may be responsible for the coordinated acclimation of leaves across the rosette that could confer resistance to stress within the duration of a natural day length.

### The influence of repeated, transient HL SAA on acclimation in existing leaves

**Figure 7:**
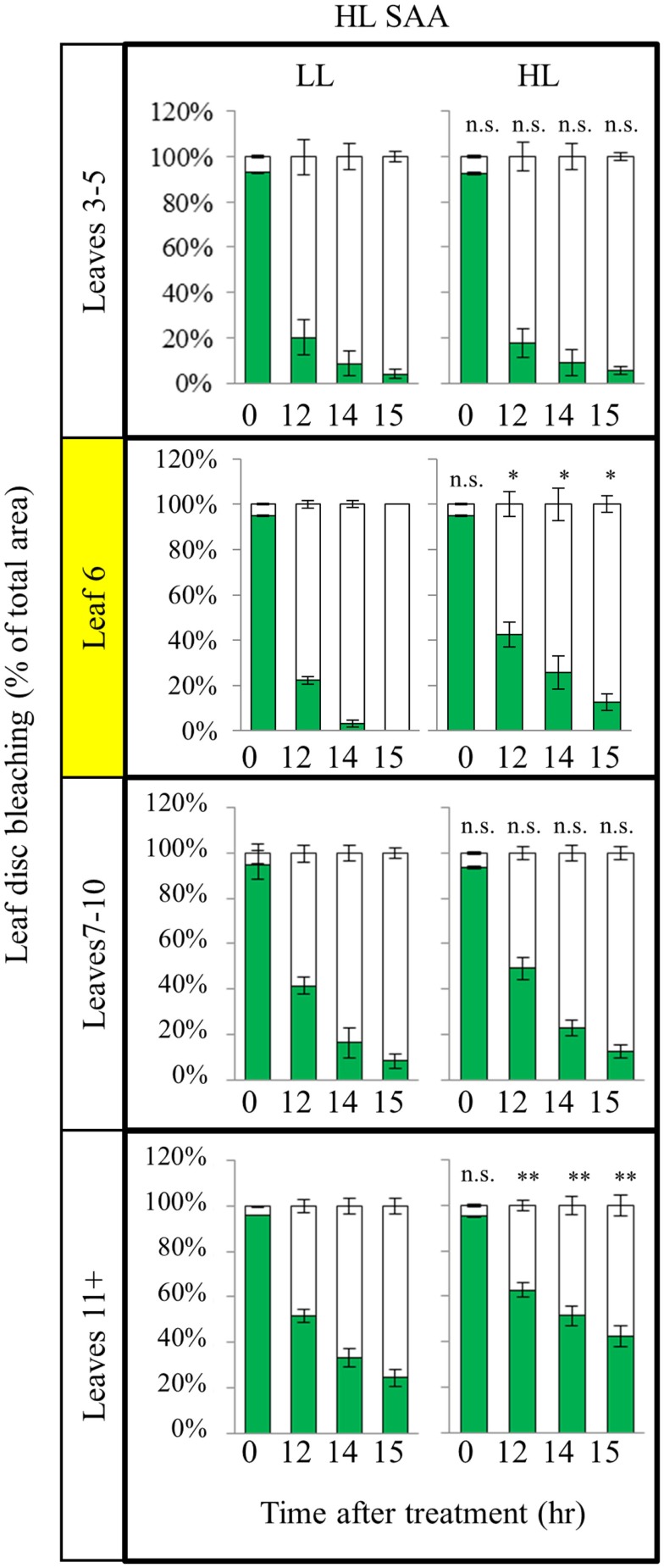
***In vitro* photo-oxidative stress tolerance assay of repeated, transient HL SAA**. *Arabidopsis* plants were either HL SAA treated for 60 min three times a day for 8 days (HL) or remained untreated (LL). Leaf disks were then taken from all leaves, floated on H_2_O_2_ and assayed as in Figure 5. Photographs were analyzed using ImageJ software, as in Figure 5, to calculate the percentage of healthy (green) and bleached (white) tissue. Pairwise *t*-tests were performed comparing the extent of bleaching between LL samples and the respective HL-treated leaves, *n* = 3.

As plants exposed to short term HL SAA treatments failed to generate a strong acclimation response, we hypothesized that repetitive treatments are required to generate stronger acclimation responses. Under long term HL conditions systemic signaling from mature leaves influences the development of new, emerging tissues mediating changes in leaf structure and thickness, chloroplast prevalence, and growth rates (Coupe et al., 2006; Araya et al., 2008; Jiang et al., 2011). However, how existing leaves respond to repeated, short term HL spot treatments in distal and exposed leaves is unknown. Plants were subject to three, 1 h HL spot treatments per day for 8 days (Figure 7). Interestingly, analysis of HL SAA treated plants showed that the exposed leaf (6) and young emerging leaves (11+) of the HL-treated plants exhibited a statistically significant increased tolerance to oxidative stress after repeated, transient stress than their respective LL controls (Figure 7). By contrast, leaves 3–5 and 7–10 showed no significant difference between the respective HL-treated and non-treated tissues.

**Figure 8:**
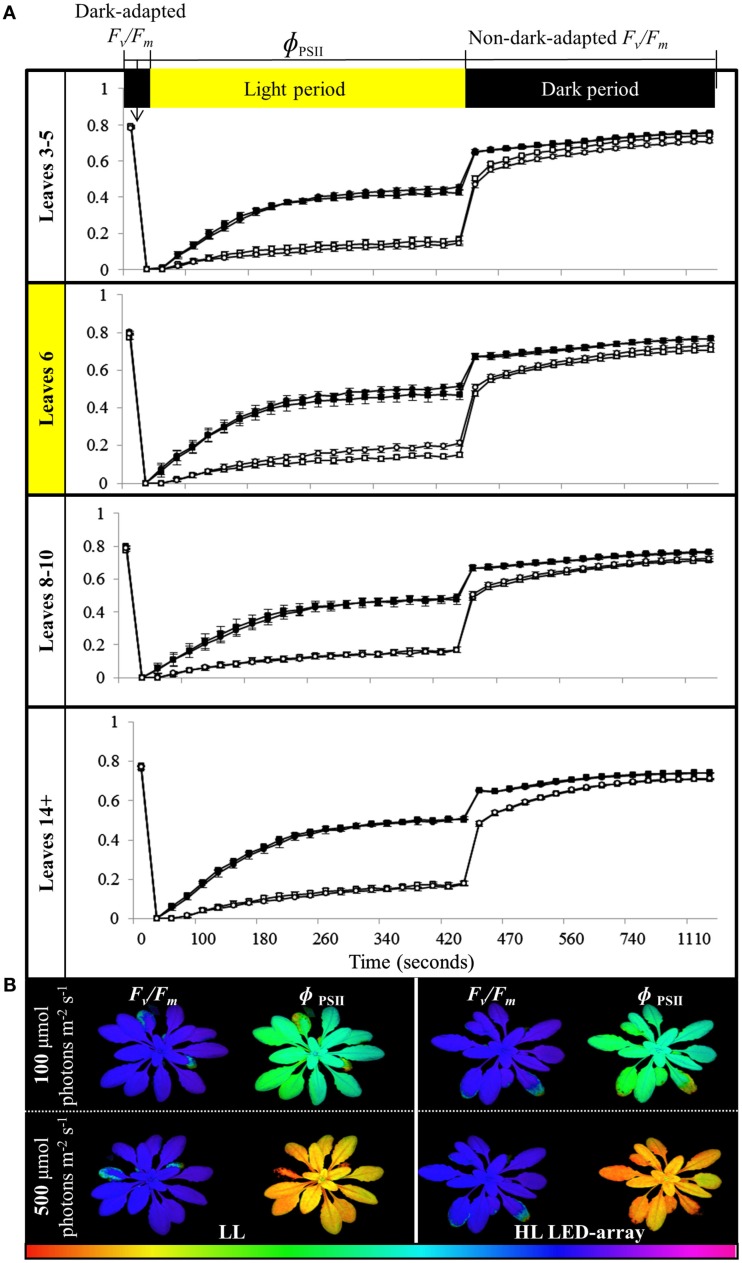
**Comparison of photoinhibition and recovery in plants after the repeated, transient HL SAA acclimation experiment exposed to 150 and 500 μmol photons m^−2^ s^−1^**. Treated plants were subjected to three separate 1 h HL LED-spot treatments per day for 8 days prior to measurement. **(A)** *F_v_/F_m_* and Φ_PSII_ measurements of both HL LED-array treated (circle) and untreated plants (square) subsequently exposed to either 150 (closed symbol) or 500 μmol photons m^−2^s^−1^ (open symbol) and dark for the indicated time are shown. The leaf position is indicated. The entire experiment was performed in triplicate, one representative is shown for which *n* = 3. **(B)** A representative false colour image of *F_v_/F_m_* and Φ_PSII_ (measured at 420 s) from HL LED-array exposed (HL) and untreated (LL) plants under 100 and 500 μmol photons m^−2^s^−1^. The colored scale bar represents the corresponding value of Φ_PSII_ or *F_v_/F_m_*, increasing in value from left (red) to right (pink).

**Figure 9:**
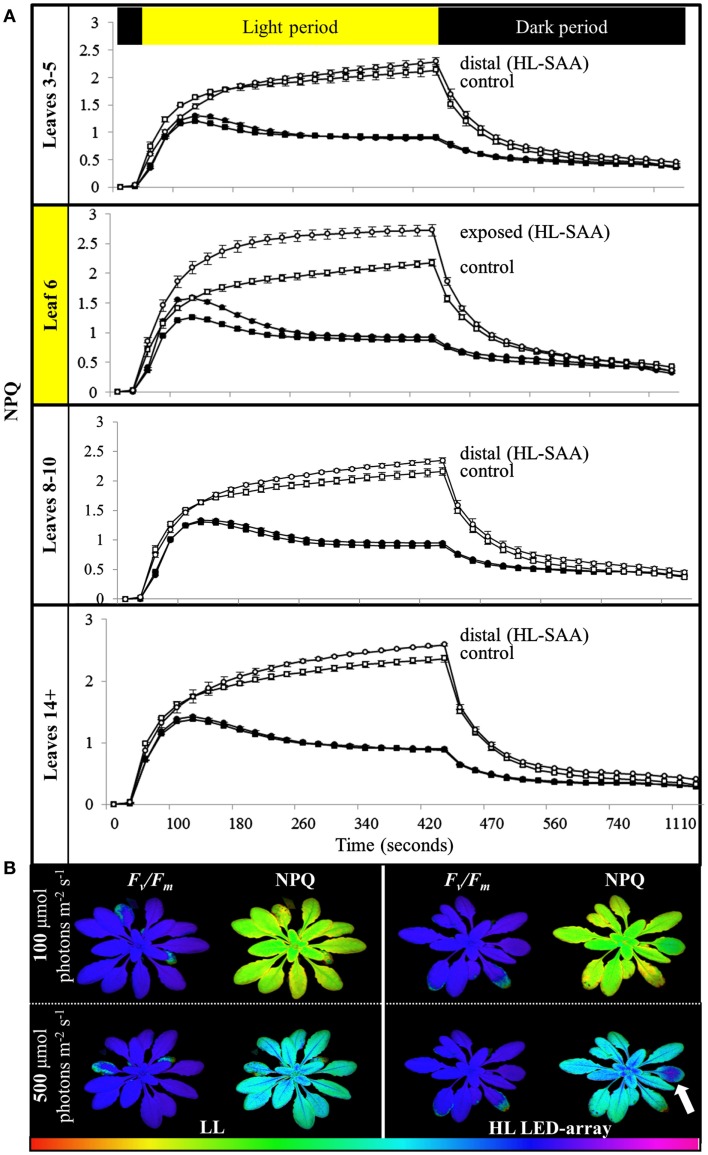
**Non-photochemical quenching induction and relaxation in plants after repeated, transient HL SAA**. Treated plants were subjected to three separate 1 h HL LED-spot treatments per day for 8 days prior to measurement. **(A)** NPQ measurements of both HL LED-array treated (circle) and control plants (square) subsequently exposed to either 150 (closed symbol) or 500 μmol photons m^−2^s^−1^ (open symbol) and dark for the indicated time are shown. The leaf position is indicated. The entire experiment was performed in triplicate, one representative is shown for which *n* = 3. **(B)** A representative false color image of NPQ (measured at 420 s) from HL LED-array exposed (HL) and untreated (LL) plants under 100 and 500 μmol photons m^−2^s^−1^. The colored scale bar represents the corresponding value of NPQ increasing in value from left (red) to right (pink). The circle of dark blue on the treated leaf six is indicated with an arrow.

To determine if the acclimation response to repeated 1 h HL treatments was also reflected in changes to photosynthesis and photoinhibition, two photosynthetic parameters, Φ_PSII_ (Figure 8) and NPQ (Figure 9), were measured at the end of the 8-day treatment. The measurements were undertaken at both 150 and 500 μmol photons m^−2^ s^−1^. Under both light intensities, all leaves from HL-exposed and untreated plants exhibited relatively similar Φ_PSII_ values (Figure 8), except for leaf 6 of the HL-exposed plants, which had slightly increased levels of Φ_PSII_. On the other hand, NPQ was markedly higher in the exposed leaf 6 and significantly higher in distal (HL SAA) tissue than in controls for the younger leaves (Figure 9). These observations indicate that repeated transient HL SAA treatments result in long term acclimation to HL in both exposed and distal leaves.

### HL SAA and auxin

**Figure 10:**
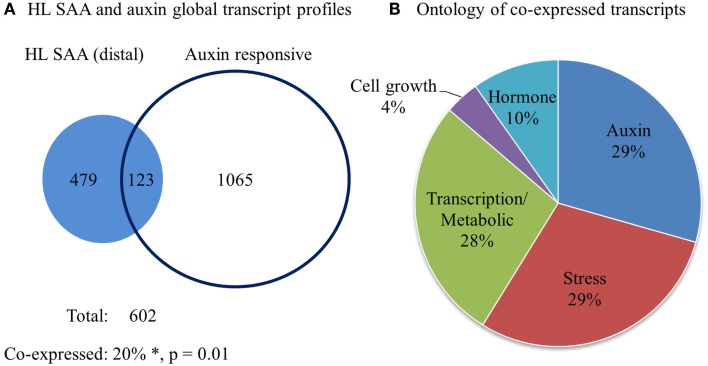
**Comparative analysis of HL SAA (distal) and auxin-responsive genes**. **(A)** Venn diagram of all transcripts that exhibit significant co-expression between 60 min HL SAA treatment microarray data (Rossel et al., 2007) and at least two of the following auxin arrays (Sawa et al., 2002; Zhao et al., 2003; Redman et al., 2004; Overvoorde et al., 2005; Nemhauser et al., 2006; Lee et al., 2009). *The percentage of co-expressed genes is greater than would be expected by chance by a two-sample *z*-statistic (equals 15.6, equivalent *p* = 0.01). **(B)** GO annotation (TAIR 10, 2012) of co-expressed genes between HL SAA and two or more auxin treatment experiments.

Our initial analysis of different HL SAA marker transcript levels demonstrated specific distal expression of *GH3.3* (Table 1), an important gene in regulating auxin homeostasis (Staswick et al., 2005). This may indicate connections between HL SAA and developmental processes mediated by auxin. To determine the influence of HL SAA on auxin-regulated transcripts, we compared the genes that exhibited significant changes in the distal leaves of HL SAA plants (Rossel et al., 2007) with data from six different auxin treatment studies (Sawa et al., 2002; Zhao et al., 2003; Redman et al., 2004; Overvoorde et al., 2005; Nemhauser et al., 2006; Lee et al., 2009). The analysis revealed that a subset of 123 (out of 602) SAA transcripts were co-expressed with auxin-responsive genes (total of 1188; Figure 10; Table 2). This was a significantly higher overlap of genes than expected by random chance (two-sample *z*-statistic = 15.6, equivalent *p* = 0.01). Using GO annotation (TAIR 10, 2012) it became evident that the co-expressed genes in both HL SAA and two or more auxin treatment experiments exhibited a large proportion of genes involved in either auxin-related (29%) or plant stress processes (29%).

**Table 2:** **Real-time RT-PCR primers and Universal ProbeLibrary probes (Roche) used for quantitative transcript analysis**.

Target sequence anotation	Gene locus	Universal Probe Library probe	Primer sequences	
*ACS2*	AT1G01480	80	F	CGACGACTTTACGAGGATGG
			R	GCTCGGAGAAGAGGTGAGTG
*AGP2*	AT2G22470	15	F	GGTTGCTTCTCCTCCTCAGA
			R	TGGAGTTAATCCAGCGGAAG
*ANOCO19*	AT1G52890	143	F	CAACAACGGTACTTCGTCCA
			R	TTGTCGATCTCTTGATGAAACG
*APX2*	AT3G09640	10	F	TCATCCTGGTAGACTGGACAAA
			R	CACATCTCTTAGATGATCCACACC
*ATBETA AMY*	AT4G15210	115	F	CCCGTTTACGTTATGCTTCC
			R	ACGTTTAAGCTGCGTTTCAAG
*BCB*	AT5G20230	3	F	GTAGGCGACGAGCTCGAAT
			R	TTCTGATACAACTGCCACATCA
*CAM5*	AT2G27030	103	F	TGTCAAAGTTATGATGGCAAAGA
			R	GAATTGCTACTACGCTTTGCTG
*CAT2*	AT4G35090	25	F	TCTGGTGCTCCTGTATGGAA
			R	TGGTAATCCTCAAGAAGGATAGGA
*CYCLO*	AT2G29960	103	F	GGCAGTTCCTAAAACTGCAGAA
			R	TTCCCTTGTAGTGTAGAGGTTTCC
*DDF1*	AT1G12610	12	F	CGGAGATGAGGCCTAAGAAG
			R	TGCCTCTGTAAACTGGGTGA
*DREB2A*	AT5G05410	121	F	GATTTTCAAATTTCGTCCCCTA
			R	TGTTCTGTTTCTATCTCCACTCTGA
*DTA1*	AT1G02400	141	F	TCATGATGATCCTTTCAAGTTCAG
			R	CCAAATCTCTAACCGTGCGTA
*EDL3*	AT3G63060	28	F	ATTGTCCGGCGAAGATCC
			R	CAGAAGAACATGAGTTTCGCTAAC
*ELIP1*	AT3G22840	126	F	GCACAAAGTTTAGCGACTTGC
			R	CGCAACGAATCCAACCAT
*ELIP2*	AT4G14690	101	F	CCACCACAAATGCCACAG
			R	GCAAATCTCCAAACTTCGTACTC
*ERF11*	AT1G28370	82	F	CGTCAAAACCAACGAAGGTAA
			R	ACGTCCCCATGGTCTCTTC
*ERF2*	AT5G47220	82	F	TTACGGAGACGGCAGTGAA
			R	AATTTCCCCCACGGTCTCT
*EX1*	AT4G33630	116	F	AGAAAGAGAAGAAGATTTCTGTCAAGA
			R	ATTTTGTCAAACCCGACAGC
*GH3.3*	AT2G23170	148	F	CATCACAGAGTTCCTCACAAGC
			R	GTCGGTCCATGTCTTCATCA
*GH3.5*	AT4G27260	148	F	CATCTCTGAGTTCCTCACAAGC
			R	GGAACGAACTGGCTCATCA
*GPX2*	AT2G31570	91	F	CCTGATGGCAAGGTCTTACAG
			R	GCAGTTTGAATGTCCTTCTCG
*GST6*	AT2G47730	15	F	AAGCAAGAGGCCCACCTT
			R	TCTTGACTCGAAAAGCGTCA
*GST24*	AT1G17170	12	F	AGACTTGGCCCGACAATAAC
			R	TCCTTCTCGCCGTAACATTC
*LHCB1.2*	AT1G29910	110	F	CCCATTGGGTCTTGCTACC
			R	CCGTTCTTGAGCTCCTTCAC
*MYB44*	AT5G67300	98	F	ACCTTCCGTTGAGCTTTTCA
			R	AGGAAGCGGTAGCACAACAG
*NCED3*	AT3G14440	22	F	TCGTCGTGATAGGGTCCTG
			R	TTCTCGTCAGACTCGTTGAAAA
*NHL10*	AT2G35980	24	F	GCCTTCTACGGTCCATCAGT
			R	GTGCCCACGTCGGTAGTAG
*NRT1.8*	AT4G21680	47	F	TGTGCACCATGAAGAGTTGAA
			R	TGTAACAATAGCAGCTCTATCCAAG
*PIN3*	AT1G70940	59	F	TCTTGGAATGGCAATGTTTAGTT
			R	CTAACCGCCATGGCAAAC
*PIN4*	AT2G01420	159	F	TGCCCAAAATATTACAACAATCC
			R	TGGGTTGAAGTGCCATGA
*PIN7*	AT1G23080	159	F	TGGGCTCTTGTTGCTTTCA
			R	TCACCCAAACTGAACATTGC
*PP2A*	AT1G13320	29	F	GACCGGAGCAACTAGGAC
			R	AAAACTTGGTAACTTTTCCAGCA
*PR2*	AT3G57260	111	F	GCTTAGCCTCACCACCAATG
			R	CCCGTAGCATACTCCGATTT
*PR6-like*	AT2G38870	155	F	GACGAGTCGTGGTTGGTTAGT
			R	AACTTTAGGCATCAGTAACACAGAAA
*Putative calcium binding protein*	AT1G21550	63	F	GATGTGTTGGAACGGCTAGG
			R	CATTCTCCCACAATCCCAAG
*Putative zinc finger protein*	AT2G42360	35	F	AAACCAGGCTGAACTTGACTG
			R	CCGGGGATACAACTGTTTTG
*RAP2.6*	AT1G43160	140	F	GGACGATGGGTCATAAGAGAGA
			R	TGAGCTTTCACATTCTTTAGTCACA
*RBCS1A*	AT1G67090	8	F	CGCTCCTTTCAACGGACTTA
			R	AGTAATGTCGTTGTTAGCCTTGC
*RD29a*	AT5G52310	69	F	ACGTCGAGACCCCGATAAC
			R	CAATCTCCGGTACTCCTCCA
*RRTF1*	AT4G34410	68	F	TCGGGTATGCATTATCCTAACA
			R	AAGCTCTTGCTCCGGTGA
*TCH4*	AT5G57560	6	F	GCTCAACAAAGGATGAGATGG
			R	CCTCTTCGCATCCGTACAAT
*UGT76E12*	AT3G46660	138	F	TCTTTGGTTACCACTCTCTAACAAGA
			R	CTCTTCGTCACAACATGTGAATC
*UGT74E2*	AT1G05680	29	F	TGTGTGGAAGGTTGGGGTA
			R	TCTTCTCTTCTCACAAACCCATC
*Unknown (F26K10.20)*	AT4G28140	143	F	TCGTCCTAAACCCTATTTCCAA
			R	AAAGGGAAAGCCTCTAACGAA
*Unknown (MFB16.16)*	AT5G50760	152	F	CAAAAGGAAAGCCGAAGAAA
			R	GGACCAACGTAAACCGTGAA
*WRKY28*	AT4G18170	70	F	AGGACGGCAGCTTATACTAACG
			R	CACTTTGTCCATATCCATAATCCA
*ZAT6*	AT5G04340	8	F	CTCGCGACGGAGATAGAAAC
			R	AAGCAGAGGAGGTGAAGACG
*ZAT10*	AT1G27730	31	F	GGACAAAGGGTAAGCGATCTAA
			R	AGAAGCATGAGGCAAAAAGC
*ZAT12*	AT5G59820	103	F	CCCACGGTGACTACGTTGA
			R	TCAAATTGTCCACCATCCCTA

**Figure 11:**
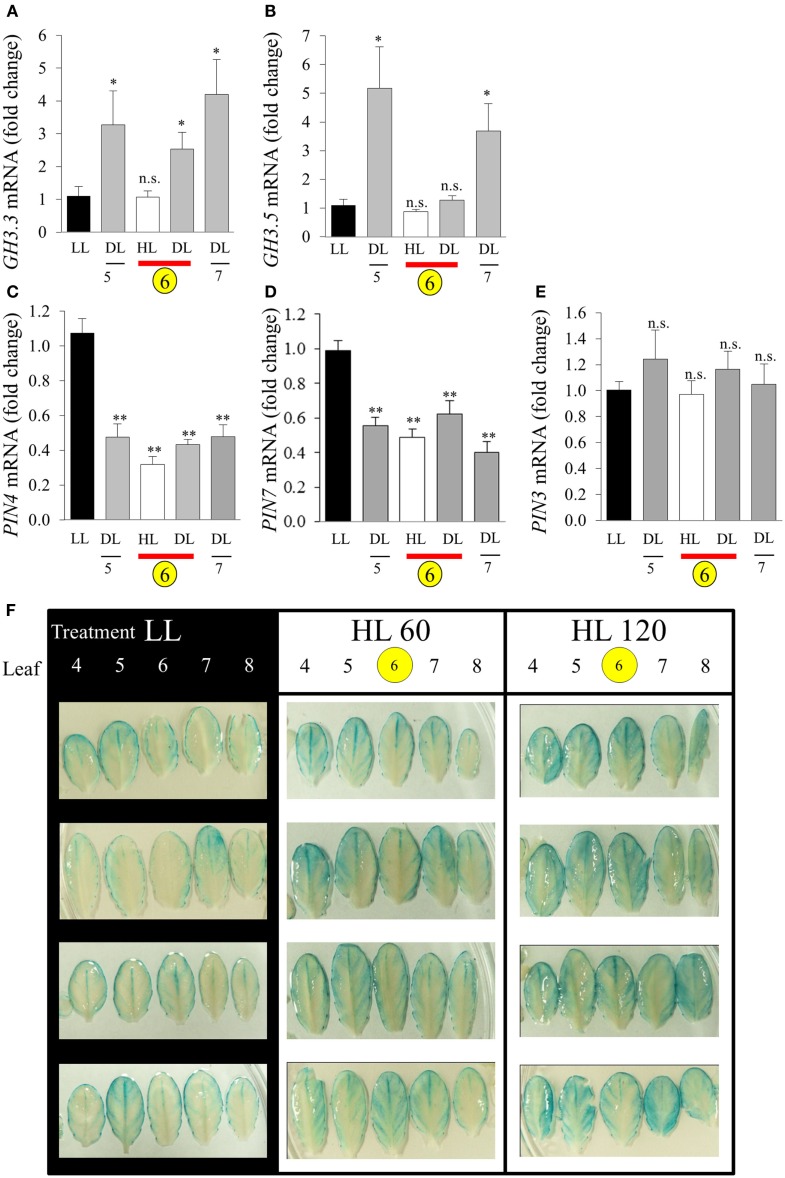
**Analysis of *GH3 a*nd *PIN* transcript accumulation during HL SAA**. Relative transcript levels of **(A)** *GH3.3*, **(B)** *GH3.5*, **(C)** *PIN4*, **(D)** PIN7, and **(E)** *PIN3* after the HL SAA treatment of leaf 6 (yellow circle). Distal tissue was sampled from leaves 5, 6, and 7 (grey), distal tissue was also sampled from within the HL-treated leaf (red bar), **(F)** Localization and distribution of auxin visualized by DR5:GUS after HL SAA. Representative images from four different plants showing leaves 4–8 (left to right) from *DR5:GUS* transgenics following illumination with either LL conditions (40 ± 25 μmol photons m^−2^s^−1^), HL LED-array treatment of leaf 6 (1500 ± 50 μmol photons m^−2^s^−1^) for either 60 (HL 60), or 120 min (HL 120). Pairwise *t*-tests were performed comparing the transcript levels in HL and DL samples with those of LL samples yielding *p*-values as shown Error bars indicate standard error, for each sample type, *n* = 6, *p < 0.05, **p < 0.001, n.s., not significant. *n* = 8 Per leaf for two independent auxin experiments.

The connection between HL SAA and auxin was further investigated by analyzing the expression of auxin-responsive genes and the spatial distribution of auxin. Five transcripts were chosen (*GH3.3*, *GH3.5*, *PIN-FORMED3 (PIN3)*, *PIN4*, and *PIN7*). After the LED-spot treatment, independent samples *t-*tests show that both *GH3* transcripts exhibited statistically significant induction in the distal leaves (Figures 11A,B). The induced expression of the *GH3* transcripts is also specifically limited to that of the distal tissues, as LSD tests on one-way ANOVAs combining all tissues show significant differences between LL, DL, and HL-treated tissues (*P* < 0.05). Whereas *PIN4* and *PIN7* were down-regulated in HL and distal tissues and *PIN3* exhibited no significant changes in transcript levels (Figures 11C–E). Auxin distribution was inferred by using the auxin-responsive DR5:GUS transgene. Under LL, plants exhibited typical *DR5:GUS* staining, mainly localized to the leaf borders, hydathodes, and main vascular tissues (Figure 11F). In contrast, after HL spot treatment the distal leaves showed increased distribution of *DR5:GUS* in secondary vasculature and mesophyll cells (Figure 11F).

## Discussion

In this study we shed light on the processes which govern the initiation of HL SAA and retrograde signaling and provided evidence for acclimation in treated and young, distal leaves that include changes to photo-oxidative stress tolerance, NPQ, and auxin-responsive gene expression in response to repeated 1 h HL treatments.

### HL SAA transcriptional response and signal initiation

Different lengths of HL treatment revealed that the induction of HL-responsive genes is abolished after 120 min, even under light stress (Figures 2A,B), highlighting the transient nature of the response to short term HL treatments. HL SAA induction was also proportional to light intensity (Figures 2C,D), suggesting a direct relationship between HL SAA signaling and retrograde signaling derived from photosynthesis in the HL-treated leaf. This hypothesis is supported by previous studies which have shown that pre-treatment with the photosynthetic inhibitor, 3-(3,4-dichlorophenyl)-1,1-dimethylurea (DCMU) was able to attenuate the SAA induction of two well-known marker genes *APX1* and *APX2* (Muhlenbock et al., 2008; Szechynska-Hebda et al., 2010). However, given the disparate nature of the HL treatment systems and marker genes used in this study, further DCMU treatments with our system would have to be conducted to support this theory. We demonstrated that the signal was initiated at low increases in light intensity, not just in response to severe stress of more than 10× of normal growth light as used in earlier studies (Karpinski et al., 1999; Mateo et al., 2004; Rossel et al., 2007; Muhlenbock et al., 2008; Miller et al., 2009; Szechynska-Hebda et al., 2010). Furthermore, the induction of these transcripts in distal tissues using a small area of applied light at moderate intensity indicates that the signaling of HL stress is not an average integration of shade and light signals generated across the plant, but a response to HL in a specific portion of a single leaf.

UVA, blue, yellow and red light-exposed plants exhibited significant systemic induction of *ZAT10* and *RRTF1* transcripts (Figures 2E,F), however the increase in mRNA under these conditions is a fraction of the observed response under white HL (Figure 2C). This may reflect the impact of the specific wavelengths on the rate of photosynthesis in the treated leaf (McCree, 1972), but would require additional photosynthetic measurements for confirmation. That is, while the intensity was the same for all treatments as in this study (1500 ± 50 μmol photons m^−2^ s^−1^), the narrow wavelength would result in a lower overall total photosynthetic available radiation (PAR) available for capture by chlorophylls and carotenoids. Interestingly, for both UVA and blue light the lack of systemic response is in despite of an observed strong physiological photobleaching response in the treated tissue (data not shown). This indicates the presence of separate retrograde signaling systems which activate HL SAA independent from those that govern blue light responses and photodamage acclimation responses (Franklin and Whitelam, 2004; Suetsugu and Wada, 2007; Takahashi et al., 2010; Lehmann et al., 2011). This is further supported by the observation that the white LEDs do not produce any UV spectra yet exhibit the highest induction of HL SAA and that HL SAA induction occurs at intensities as low as 250 μmol photons m^−2^s^−1^. Such intensities are unlikely to even induce the xanthophyll cycle as zeaxanthin typically accumulates in response to 400 μmol photons m^−2^s^−1^ and above (Demmig-Adams et al., 1989). Thus, the induction at 250 μmol photons m^−2^s^−1^ indicates that changes in photosynthetic parameters and subsequent retrograde signals initiate HL SAA, not oxidative stress and damage, although the latter may contribute to the intensity of the response at higher light intensities.

Interestingly, blue light treatments resulted in increased transcript induction for both *RRTF1* and *ZAT10* compared to the other wavelengths (Figures 2E,F). This may be attributed to the known role of blue light in multiple acclimation responses (Liscum and Briggs, 1995; Folta and Spalding, 2001; Jarillo et al., 2001; Danon et al., 2006; Matsuda et al., 2008). Contrary to this hypothesis, it was shown that under HL the systemic induction of *APX1* and *APX2* exhibit no apparent attenuation and dependency of blue light perception via cryptochrome photoreceptors in double mutants *cry1*/*cry2* (Szechynska-Hebda et al., 2010). The discrepancy between the analyses and results presented in this study highlight the complexity of HL SAA and possible involvement of different retrograde signals including ROS and photosynthesis-mediated signaling pathways. Two major forms of ROS, H_2_O_2_ and ^1^O_2_, trigger different transcriptional responses including induction of *ZAT10* and *RRTF1*, respectively (op den Camp et al., 2003; Gadjev et al., 2006). Under HL SAA the downstream transcriptional regulation of *APX* isoforms may be influenced by both H_2_O_2_-derived and blue light ^1^O_2_-derived signaling and be dependent on how these different ROS signaling pathways interact.

### The influence of temperature and humidity on HL SAA

Even though heat exposure is able to cause photoinhibition (Allakhverdiev et al., 2008; Sainz et al., 2010) and to be synergistic to photo-oxidative stress (Rossel et al., 2002; Mittler, 2006), our study reveals that moderate heat stress does not influence HL SAA induction. That is, the increase in induction of *RRTF1* and *ZAT10* in distal leaves was similar at all analyzed temperatures (Figure 3), although at 32°C there was a suppression of *RRTF1* transcripts relative to 21°C in all three tissues, including the control.

In contrast to increased temperatures lower RH levels proportionally inhibited HL SAA induction in distal leaves (normalized to LL 90% RH; Figure 3B). This is surprising as 70% of HL inducible genes are also induced by drought stress and there are common regulators of both pathways that alter the expression of *ZAT10* and *APX2*, such as *SAL1* (Kimura et al., 2003; Wilson et al., 2009; Estavillo et al., 2011). Furthermore, low humidity is already known to induce *APX2* (Karpinski et al., 1997; Fryer et al., 2003; Hetherington and Woodward, 2003; Szechynska-Hebda et al., 2010); that is if anything a synergistic or additive effect of low RH and HL may have been expected. Yet, there was no additive induction in distal leaves at lower RH. This either reflects an epistatic effect, or the lower RH impairs the propagation of the SAA signal to distal leaves. With respect to epistasis, as noted above, both drought and HL have similar impacts on ABA induction and expression of genes such as *APX2* and *ZAT10* (Rossel et al., 2006) and it could be low RH and HL SAA act via the same pathway. Contradictory to this, there is an additive increase in gene expression in HL-treated leaves at low RH (Figure 3B). Furthermore, the drought and HL stress signaling SAL1 mutant, *alx8*, also retains the additive increase in *APX2* and *ELIP2* gene expression under drought and light stress (Rossel et al., 2006; Estavillo et al., 2011). This suggests that the loss of HL SAA induction under low RH is more consistent with impaired propagation than epistasis. This is intriguing as HL SAA acts via the vasculature, but most likely not in the xylem as it is observed in upper and lower leaves (Figure 5). Proposed SAA signals include ROS and electrochemical gradients, none of which are directly impacted by changes in transpiration. How exactly low RH impacts HL SAA signaling still needs further investigation.

### The response to repeated, short term, localized HL

To date, the study of acclimation processes and function of HL SAA has been restricted to evaluation of the immediate adaptation responses to one or several hours of HL (Rossel et al., 2007; Muhlenbock et al., 2008; Szechynska-Hebda et al., 2010). However, our study demonstrates that the single application of a highly localized signal did not result in any observable distal acclimation *in vitro* beyond the transcriptional changes (Figures 4–6). This is in contrast to earlier reports using 1/3 rosette HL treatment that resulted in distal acclimatory changes with respect to H_2_O_2_ tolerance and NPQ (Karpinski et al., 1999; Rossel et al., 2007; Szechynska-Hebda et al., 2010). Significantly, repeated, short term applications of the HL spot treatment over 8 days resulted in enhanced tolerance to H_2_O_2_ and elevated NPQ capacity compared to LL controls (Figures 7–9). Key to these observations was that the acclimatory response was increased in younger leaves as they had lower levels of *RRTF1* and *ZAT10* mRNA accumulation (Figure 7), and higher basal resistance to H_2_O_2_ bleaching compared to LL control plants (Figure 5). Younger leaf tissues are already described to exhibit increased resistance to numerous other stress conditions including salt, drought, temperature, and ROS (Takagi et al., 2003; Jung, 2004; Muhlenbock et al., 2008; Hajlaoui et al., 2010; Yoon et al., 2011). The processes governing their acclimation in response to stress, however, are unclear (Takagi et al., 2003; Jung, 2004; Yoon et al., 2011).

Auxin is a well-established regulator of many plant processes including organ patterning, root and shoot architecture, vascular development, growth, and tropic responses (Benjamins and Scheres, 2008; Zhao, 2010). Our study describes how HL SAA is able to regulate distal-specific auxin-related gene transcription as well as free auxin distribution (Aloni et al., 2003) in both HL-treated and distal tissues (Figure 11). The *GH3.3* and *GH3.5* were exclusively expressed in distal tissue in response to HL SAA (Figure 11) and are from a class of proteins directly responsible for the maintenance of auxin homeostasis (Staswick et al., 2005). Over-expression of *GH3.5* alters the balance between free and conjugated auxin enhancing tolerance to pathogen infection and abiotic stresses such as drought, salinity, and temperature (Park et al., 2007; Zhang et al., 2007). Even though *GH3.3* is induced under pathogen infection its role in plant stress is relatively undefined (González-Lamothe et al., 2012). The proposed integration between auxin, oxidative stress, and ROS was reviewed recently (Tognetti et al., 2012). Auxin is capable of influencing ROS homeostasis by regulating proteins involved in ROS detoxification, including transcription regulators, the DELLA proteins and the ROS detoxifying enzymes, glutathione *S*-transferases (Laskowski et al., 2002; Paponov et al., 2008). Conversely, ROS produced under various stress conditions greatly influences auxin biosynthesis, metabolism, transport, and signal transduction pathways in exposed tissues (Tognetti et al., 2012). It is now evident that distal tissues of plants subjected to repeated HL spot treatments may exhibit similar changes in auxin-mediated processes.

In conclusion localized HL treatments and repeated, localized HL treatments initiate retrograde signals that lead to transcriptional and acclimatory responses in both treated and distal tissue. However, a single 1 h HL spot treatment is not sufficient to alter the acclimation response in distal tissues. HL SAA requires either a 1/3 of the rosette to be treated (Rossel et al., 2007), or a single leaf to be repeatedly subject to 1 h HL treatments. Questions remain as to whether the response to repeated HL SAA is at the cellular or subcellular level? What is the nature of the memory of repeated HL, is it for example due to changes in chromatin? Is the response reversible and does auxin contribute directly to the acclimation response? Why do young and old leaves respond differently to HL SAA? The nature of the signal and the respective roles of auxin and oxidative stress responsive genes in HL SAA from a temporal perspective, all require further investigation.

## Conflict of Interest Statement

The authors declare that the research was conducted in the absence of any commercial or financial relationships that could be construed as a potential conflict of interest.

## Footnotes

^1^http://www.philipslumileds.com/

^2^http://rsbweb.nih.gov/ij/
